# Dr. Mira Sen (Banerjee)

**Published:** 2010

**Authors:** Ashok Banerjee

**Affiliations:** Ex. Prof. of Surgery, C.N.M.C. Kolkata P1, C.I.T Road, Scheme VI M(S) Kolkata 700054 Tel: 91-9831541256 / 033 23628418 Email: asokban@vsnl.net

Dr. Mira Sen (Banerjee) was born on July 20, 1934 in Dhalaghat village of Chittagong district of East Bengal (Bangladesh), in a well-educated and cultured family. Her father, Sri Ramani Ranjan Sen, was a professor of history in the famous Braja Mohan College of Barisal. Her mother, Sushila Bala Sen, was an accomplished lady of great dignity and discipline and a constant source of inspiration to the family. Dr. Sen grew up in Barisal with her elder sister, and two brothers, one older and one younger to her.

She did her schooling from Municipal Primary Girls School at Barisal. She appeared for her matriculation from Sadar Girls High School in 1948 and passed in first division under the East Bengal Secondary Education Board, Dacca, in 1948. She completed her intermediate in science in 1950 from City College, Calcutta. During the same year, she took admission for B.Sc. Physics (Hons) at Scottish Church College, Calcutta.

In 1951, she discontinued her B.Sc. to join MBBS degree course at the National Medical College. She was the class assistant in surgery while in college. Dr. Sen passed her MBBS exam in 1956 with honours in four subjects (special honours marks were given then based on performance) and was awarded a gold medal for the same. She was also awarded the Gold Medal for Proficiency in Medicine by National Medical College, Calcutta.

Dr. Sen started her professional career in 1959 under West Bengal Health Services with her posting in NRS Medical College as demonstrator in the Department of Anatomy. In 1961, she was granted paid study leave and proceeded to the UK to join the Royal College of Edinburough. After obtaining the FRCS degree in 1964, she came back and in 1965, Dr. Sen joined as RS General Surgery at Medical College Calcutta. In 1968, Dr. Sen got married to the eminent surgeon, Dr. Asok Banerjee. She was later transferred to SSKM hospital as Lecturer in Plastic Surgery. Eventually, Dr. Sen became the Professor and HOD of Plastic Surgery at SSKM. Later, she was transferred to National Medical College and retired as HOD and Professor of Plastic Surgery from that Institution on 31 July 1994.

She has to her credit a large number of papers published in reputed medical journals. Dr. Sen was a dedicated and revered teacher and research guide for her students. She was well known as an examiner for her fairness and impartiality and was M.Ch. examiner for various universities, e.g., Calcutta, Patna, Benares, Osmania and many others. She was also an examiner for the D.N.B. examinations.

Being a woman of determination and dedication, she continued her work as an honorary visiting surgeon at Mission Hospital for Women and Calcutta Heart Hospital. In order to keep abreast with the current developments in this emerging field, she continued with her private professional practice as well.

Dr. Mira Sen will be remembered for a very long time by her colleagues, students and members of the Association of Plastic Surgeons of India.

**Figure 1:**
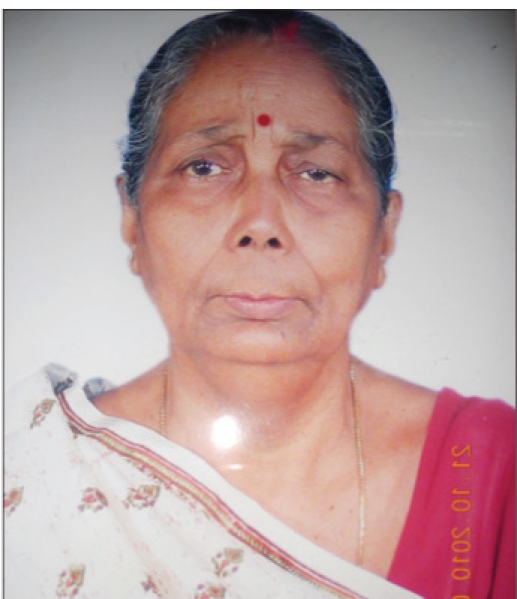
Dr. Mira Sen (Banerjee) (1934–2009)

